# Natural Infection of *Aedes aegypti* by *Chikungunya* and *Dengue type 2 Virus* in a Transition Area of North-Northeast Brazil

**DOI:** 10.3390/v11121126

**Published:** 2019-12-05

**Authors:** Carine Fortes Aragão, Valéria Cristina Soares Pinheiro, Joaquim Pinto Nunes Neto, Eliana Vieira Pinto da Silva, Glennda Juscely Galvão Pereira, Bruna Laís Sena do Nascimento, Karoline da Silva Castro, Ariadne Mendonça Maia, Clistenes Pamplona Catete, Lívia Carício Martins, Wanderli Pedro Tadei, Sandro Patroca da Silva, Ana Cecília Ribeiro Cruz

**Affiliations:** 1 Programa de Pós-Graduação em Biologia dos Agentes Infecciosos e Parasitários, Universidade Federal do Pará, Belém, PA 66075-110, Brazil; carinefaragao@gmail.com; 2 Laboratório de Entomologia Médica, Centro de Estudos Superiores de Caxias, Universidade Estadual do Maranhão, Caxias, MA 65604-380, Brazil; vc_pinheiro@hotmail.com; 3 Seção de Arbovirologia e Febres Hemorrágicas, Instituto Evandro Chagas, Secretaria de Vigilância e Saúde, Ministério da Saúde, Ananindeua, PA 67030-000, Brazil; joaquimneto@iec.gov.br (J.P.N.N.); elianapinto@iec.gov.br (E.V.P.d.S.); bioagl2013@gmail.com (G.J.G.P.); brunanascimento@iec.gov.br (B.L.S.d.N.); liviamartins@iec.gov.br (L.C.M.); spatroca@gmail.com (S.P.d.S.); 4 Programa de Pós-Graduação em Biologia Parasitária na Amazônia, Universidade do Estado do Pará, Belém, PA 66087-670, Brazil; karolflu@hotmail.com (K.d.S.C.); ariadnemaia16@gmail.com (A.M.M.); 5 Instituto Evandro Chagas, Secretaria de Vigilância e Saúde, Ministério da Saúde, Ananindeua, PA 67030-000, Brazil; clistenescatete@iec.gov.br; 6 Laboratório de Malária e Dengue, Instituto Nacional de Pesquisas da Amazônia, Manaus, CEP 69060-001, Manaus - AM, Brazil; wptadei@gmail.com

**Keywords:** *Aedes*, *Chikungunya virus*, *Dengue virus*, arbovirus

## Abstract

Dengue fever, chikungunya, and Zika are diseases caused by viruses transmitted by *Aedes aegypti* and *Aedes albopictus*. In Brazil, the number of human infections is high, but few studies are performed in mosquito vectors. This study aimed to investigate the presence of Zika, Dengue and Chikungunya viruses in *Ae. aegypti* and *Ae. albopictus* from the municipalities of Alto Alegre, Caxias, Codó, and São Mateus do Maranhão, located in the state of Maranhão, Northeast Brazil. The mosquitoes were collected with a mechanical aspirator, identified, triturated, and then submitted to RNA extraction and RT-qPCR. The positive samples were confirmed by virus isolation and genome sequencing. Three hundred and forty-eight *Ae. aegypti* (176 males and 172 females) and 12 *Ae. albopictus* (eight males and four females) were collected and tested. *Ae. aegypti* was the only vector positive in two municipalities—Codó, with detection of *Chikungunya virus* (CHIKV) belonging to the East-Central-South African genotype, and in Caxias, with detection of *Dengue virus* (DENV)-2 belonging to the Asian/American genotype. The detection of CHIKV and DENV-2 is evidence that those viruses are maintained in arthropod vectors, and shows the epidemiological risk in the area for chikungunya cases and a possible increase of severe dengue cases, associated with the occurrence of dengue hemorrhagic fever.

## 1. Introduction

Arboviral diseases are infections caused by viruses transmitted by arthropods, reported worldwide, especially in tropical climate areas [1,2]. In recent decades, the arboviral diseases have had even greater repercussions due to the overwhelming spread and increased severity, leading to a higher number of deaths [3]. In Brazil, a predominantly tropical country, there are records of a wide variety of arboviral diseases [1,4,5,6].

Dengue is the most important arboviral disease in the country and the most widely reported. According to the Brazilian Ministry of Health, a total of 1,455,898 probable dengue cases have been reported in the country from January to September 2019 [7]. The disease is caused by *Dengue virus* (DENV) (genus *Flavivirus*, family *Flaviviridae*), which has four serotypes, characterized by four to six genotypes: DENV-1 (genotypes I, II, III, IV, V, and VI) [8,9], DENV-2 (Asian/American, Asian 1, Asian 2, Cosmopolitan, and American genotypes) [10], DENV-3 (genotypes I–V) [11], and DENV-4 (genotypes I, IIA, IIB, III, IV [sylvatic], and V) [12].

The epidemiological situation in the country became even more alarming with the introduction of two other important arboviruses: *Zika virus* (ZIKV) and *Chikungunya virus* (CHIKV) [13,14,15]. The similar pathogenicity shared by those viruses resulted in difficulties in the diagnosis and clinical management [6]. In addition, congenital abnormalities, such as microcephaly and Guillain–Barré syndrome, were associated with infection by ZIKV and CHIKV [16,17,18,19,20,21,22].

ZIKV also belongs to the *Flavivirus* genus in the *Flaviviridae* family and it is phylogenetically characterized in two genotypes, African and Asian [23]. The latter was the genotype responsible for the cases occurred during the epidemic in Brazil [24]. After its introduction, the implementation of the national policy for the disease control resulted in the decrease of notifications in subsequent years. In 2017 and 2018, a total of 17,593 and 8680 cases, respectively, were reported in the country [25]. However, in 2019, a slight increase in the number of notifications (9965 cases) was reported until August [7].

CHIKV belongs to the *Alphavirus* genus, *Togaviridae* family and it is characterized by four distinct lineages: East-Central-South African (ECSA), West African, Asian, and Indian Ocean [26]. In Brazil, both ECSA and Asia lineages were introduced in 2014 [15]. 

After the co-circulation with ZIKV and DENV in 2016, a total of 185,593 chikungunya probable cases were reported in 2017, however, a decrease in cases reported was observed in 2018 (87,687 reported cases) [25]. In 2019, an increase in cases is observed in the country and, until September, 115,510 cases were reported [7].

The transmission of DENV, ZIKV, and CHIKV occurs through the bite of infected mosquitoes, and *Aedes aegypti* and *Aedes albopictus* are the most widely recognized species in the viral transmission worldwide. In Brazil, *Ae. aegypti* is incriminated as the primary vector due to its domestic urban behavior, which coincides with the most affected areas [27,28]. 

The state of Maranhão, located in Northeast Brazil, is considered one of the poorest areas of the country [29] and presents, in most municipalities, high *Ae. aegypti* infestation levels [30], which corroborates with several studies conducted in the region [31,32,33,34,35]. This scenario highlights the role of monitoring viral circulation in periods that precede the outbreaks, especially by entomovirological surveillance investigations.

Currently, entomovirological surveillance is being used worldwide, as it allows the early detection of viruses and enables the implementation of vector control strategies, minimizing the impact of the epidemics.

DENV entomological surveillance has been performed on the island of São Luís, Maranhão in the past, but no viral detection was reported [32]. Recently, CHIKV was detected in *Ae. aegypti* in two municipalities of Maranhão, Caxias and São Mateus do Maranhão [36], corroborating the clinical data of the occurrence of this arbovirus in the region. In this study, we investigated the natural infection by DENV, ZIKV, and CHIKV in *Ae. aegypti* and *Ae. albopictus* collected in urban areas with case reports in municipalities of state of Maranhão.

## 2. Materials and Methods

### 2.1. Study Area

**Figure 1:**
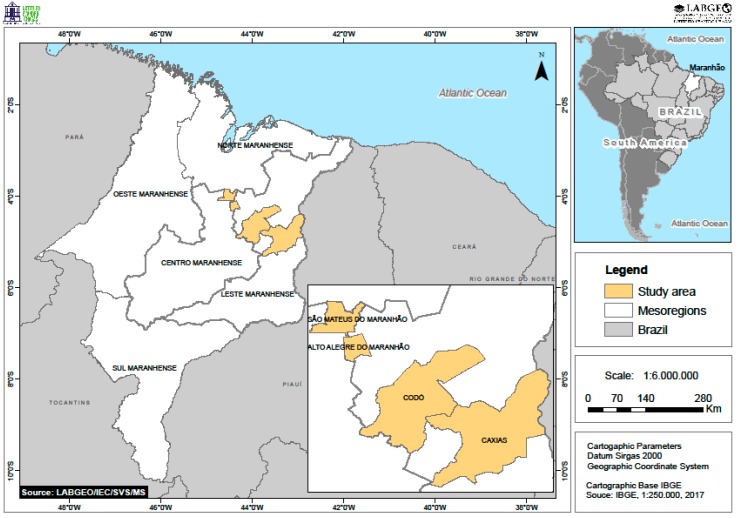
Study area with the municipalities where insect collections were performed: Alto Alegre do Maranhão, Caxias, and Codó, located in the Leste Maranhense mesoregion, and São Mateus do Maranhão, located in the Centro Maranhense mesoregion.

The state of Maranhão is located in the Northeast Region of Brazil, in a transition region between the Amazon and Cerrado biomes, and is limited to the north with the Atlantic Ocean, which provides the most diverse morphological characteristics to this area. It has 329,642,170 km^2^ of territorial extension, being the second largest state in the region and the eighth largest among the Brazilian states. An estimated population of 7,035,055 inhabitants was reported in 2018. The state is divided into five geographical mesoregions (Norte Maranhense, Oeste Maranhense, Centro Maranhense, Leste Maranhense, and Sul Maranhense), subdivided into 21 geographic microregions with 217 municipalities, including the capital, São Luís [37], as shown in Figure 1. 

### 2.2. Insect Collection

The collections, authorized by the Instituto Chico Mendes de Conservação da Biodiversidade (ICMBio) and Sistema de Autorização e Informação em Biodiversidade (SISBIO) (Nº 11965-2), were performed in four municipalities, selected due to the occurrence of arbovirus cases, clinically diagnosed and notified by the Epidemiological Surveillance of Health Secretariats: Alto Alegre do Maranhão, Caxias, Codó (located in the Leste Maranhense mesoregion) and São Mateus do Maranhão (located in the Centro Maranhense mesoregion), as shown in Figure 1.

Six collection expeditions were carried out during 12 alternated months: September and November 2016; January, March, May, and July 2017. In each municipality, three neighborhoods with the highest case notifications were selected. In each neighborhood, over one day, the collections were performed in ten residences located within two blocks. Aspirations were performed in 120 residences, resulting in 720 during the whole study period. Each collection was performed for 15 minutes, inside and around the property, using a 12 volt battery electric aspirator, similar to that designed by Nasci [38], with modifications.

The collected mosquitoes were rapidly anesthetized (about 1 min) with ethyl acetate-soaked cotton inside the sealed collection tube, then transferred to 5 mL test tubes, identified with the place and date of collection, stored at −70 °C, and transported in liquid nitrogen (−196 °C) to the Instituto Evandro Chagas (IEC) in Ananindeua, Pará, for taxonomic identification and molecular analysis.

### 2.3. Identification

The specimens were identified in the Medical Entomology Laboratory in the Arbovirology and Haemorrhagic Fevers Department, Evandro Chagas Institute (SAARB/IEC), on a refrigerated table at −20 °C, using species identification keys [39,40,41,42,43,44,45,46] to achieve the most specific taxonomic level possible. 

Only insects of medical importance of the Diptera order, belonging to Culicidae, Ceratopogonidae, and Psychodidae families, were considered for identification. *Ae. aegypti* and *Ae. albopictus* males and females were used in virological analyses. Females from other species were identified and quantified only for fauna characterization inside the residences. Female mosquitoes were segmented into head and body (which included thorax, abdomen, and annexes [wings and legs]) with the aid of individual tweezers and a stylet to avoid cross contamination for analysis of viral spread inside the mosquito. Males were analyzed entirely for the verification of transovarian viral transmission. After counting, mosquitoes with separated head and body were grouped in 2 mL microtubes in pools of up to 30 specimens, by species, sex, collection location and date, and whether they were engorged or not. This separation was used to determine if the infection was disseminated or whether it was from the blood retained in the intestine of the engorged mosquito. The pools were identified with an exclusive registration adopted in the SAARB/IEC, which is composed of the initials AR (of arthropod), followed by a sequel number.

### 2.4. Sample Preparation for Analysis

To obtain the mosquito suspension, a solution of 1 mL of Dulbecco’s phosphate buffered saline diluent 1X (D-PBS) (Life technologies, Carlsbad, CA, USA) with 2% penicillin and streptomycin, 1% fungizone, and 5% fetal bovine serum was added to each mosquito pool and then macerated on TissueLyser II equipment (Qiagen, Hilden, Alemanha) at 25 Hz for 1 min using a 3 mm diameter tungsten sphere based on an adapted protocol [47].

### 2.5. Viral RNA Extraction

After centrifugation for 10 minutes, 10,000 rpm, at 4 °C, the macerate´s supernatant was collected and viral RNA was extracted using the QIAamp viral RNA^®^ kit (Qiagen, Hilden, Germany), following the manufacturer’s guidelines. To validate the extraction process, we used a non-competitive internal control, genomic RNA of bacteriophage MS2 (Roche Diagnostics, Basel, Switzerland), which was added to the sample during extraction and, as negative control, nuclease-free water was used. Two microliters of internal control, diluted 1:1000 in nuclease-free water, were added to each sample. The extraction was validated by monitoring the amplification during the RT-qPCR using primers and probes for the MS2 target, as described previously [48]. 

### 2.6. Real Time Reverse Transcription Polymerase Chain Reaction (RT-qPCR) Assay

The RT-qPCR assay was performed using the SuperScript® III Platinum® One-Step RT-qPCR Kit (ThermoFisher Scientific, Waltham, MA, USA) and the primers and probes used for detection of DENV, ZIKV, and CHIKV [49,50,51]. The reaction was performed on the 7500 Fast real-time PCR thermocycler (ThermoFisher Scientific, Waltham, MA, USA) in a final volume of 25 μL. 

For DENV detection, a multiplex assay for identification of the four serotypes was used. The reaction consisted of 12.5 μL of mix (2 ×), 2.2 μL of nuclease-free water, 1 μL of primer forward and reverse (F/R) DENV-1 and DENV-3 and 0.5 μL of primer F/R DENV-2 and DENV-4, 0.45 μL of probe of each serotype, 0.5 μL of SuperScript III Platinum Taq enzyme mix, and 5 μL of extracted RNA. For ZIKV and CHIKV detection, singleplex assays were performed and the reaction consisted of 12.5 μL of mix (2 ×), 5.5 μL of nuclease-free water, 1.0 μL of primer F/R, 0.5 μL of probe, 0.5 μL of SuperScript III platinum Taq enzyme mix, and 5 μL of extracted RNA. The thermo cycling conditions consisted of an initial step of RT at 50 °C for 30 min, followed by a step of denaturation for 2 min at 95 °C and by 45 cycles of 15 sec at 95 °C, and 1 min at 60 °C. Positive (RNA extracted from DENV, CHIKV, and ZIKV infected mouse brains) and negative (nuclease-free water) controls were used to validate the test. Samples were analyzed in duplicates and considered positive with a cycle threshold (CT) average value lower than 37.

### 2.7. Viral Isolation

Positive samples by RT-qPCR assay were inoculated into *Aedes albopictus* clone C6/36 (ATCC: CRL-1660) cell culture to confirm infection and increase viral load for subsequent sequencing. C6/36 cells were seeded in 10 mL culture tubes containing 1.5 mL glutamine modified Leibowitz medium (L-15) with 2.95% tryptose phosphate, non-essential amino acids, antibiotics (penicillin and streptomycin), and 2% fetal bovine serum. After three days, the culture medium was discarded and 100 µL of sample supernatant was inoculated into the cell monolayers and the tubes were kept in an incubator at 28 °C for one hour for adsorption and carefully homogenized every 15 min. Then, 1.5 mL of maintenance medium previously prepared with glutamine-modified Leibowitz medium (L-15), with 2.95% tryptose phosphate, non-essential amino acids, antibiotics (penicillin and streptomycin), and 2% fetal bovine serum (FBS) were added, according to an adapted protocol [52]. Inoculated cell cultures were kept at 28 °C and were observed daily for 7 days using an inverted binocular microscope to verify the occurrence of the cytopathic effect (CPE). After that, the tubes with 100 µL of supernatant were passaged into new tubes as described previously. Each sample was submitted to three serial passages. Positive and negative controls were used to give reliability to the test. 

### 2.8. Indirect Immunofluorescence (IIF) Test

The IIF test was performed as described previously [53] with adaptations, in order to confirm the cell infection and to identify the infecting virus after isolation in cell culture. Inoculated cells were harvested and fixed in acetone on immunofluorescence slides for ten minutes at −20 °C. Subsequently, 10 µL of polyclonal antibodies for the *Alphavirus* and *Flavivirus* antigenic groups, diluted 1:20 in phosphate saline (PBS) pH 7.4, were added to each slide hole. Slides were incubated for 30 min at 37 °C and 5% CO_2_, washed in PBS pH 7.4 for ten minutes, and rinsed in distilled water. After drying, 10 μL of anti-mouse conjugate (Cappel) diluted 1:900 was added in each hole, incubated, and washed as described previously. After drying, the slides were assembled with a coverslip in buffered glycerol, observed in a fluorescence microscope (Olympus BX51, UPlanFL N 20X/0.5 objective and WB and U-25nd filters) and photographed at 40 × magnification using a Canon PowerShot G6 camera (Canon, Tokyo, Japan).

### 2.9. Nucleotide Sequencing

#### 2.9.1. RNA Extraction

After three serial passages in C6/36 cell culture, supernatants were submitted to viral RNA extraction using QIAamp® viral RNA kit (Qiagen, Hilden, Germany), following the manufacturer’s guidelines.

#### 2.9.2. Genomic Sequencing

The CHIKV and DENV genomes were recovered by synthesizing the first and second cDNA strands constructed directly from the ssRNA. The synthesis was performed using the cDNA Synthesis System kit (Roche Diagnostics, Basel, Switzerland) and 400 μM of Roche random primer. Subsequently, the products were purified using magnetic beads (Agencourt AMPure XP Reagent, Beckman Coulter, Brea, CA, USA) and three washes with 800 µL of 70% ethanol. The cDNA was eluted in 16 μL of 10 mM Tris-HCl (pH 7.5). The cDNA library was prepared and sequenced using the protocol described in the Nextera XT DNA Library Preparation Kit on a MiniSeq platform (Illumina Inc., San Diego, CA, USA). 

#### 2.9.3. Genome Assembling and Phylogenetic Analysis

The genome assembly was carried out using the *De Novo Assembler* methodology in the IDBA-UD program [54]. All the contigs were aligned and compared to the virus protein *RefSeq* database available in National Center for Biotechnology Information (NCBI) through DIAMOND [55]. The result was visualized in Megan6 software [56] and the inspection and annotations of putative open reading frames (ORF) genes were performed using Geneious v. 9.1.6 software (Biomatters, Auckland, New Zealand).

A multiple sequence alignment (MSA) was performed using the Mafft v. 7 program [57]. Before phylogenetic analysis, the ProtTest was applied to select the best-fit models of amino acid substitution [58]. The reconstruction of phylogenetic trees was performed using the maximum likelihood (ML) method [59] implemented in RaxML v. 8.2.4 [60]. For determination of the reliability of the tree topology, a bootstrap analysis [61] was carried out on 1000 replicates.

## 3. Results

### 3.1. Collection and Identification

A total of 3166 insects of medical importance were collected, as shown in Table 1, and were mainly of *Culex quinquefasciatus* (N = 2644, 83.51%). *Ae. aegypti* was the second most found species, with 348 (10.99%) specimens collected (176 males and 172 females), while 12 (0.38%) specimens were of *Ae. albopictus* (eight males and four females). Both *Aedes* species were grouped into 158 pools (153 pools of *Ae. aegypti* and five pools of *Ae. albopictus*). 

### 3.2. RT-qPCR Assay

Of 158 pools of *Ae. aegypti* and *Ae. albopictus* analyzed for detection of DENV, ZIKV, and CHIKV by the RT-qPCR test, three were positive and were from the municipalities of Codó and Caxias.

One pool composed by three bodies of engorged *Ae. aegypti* females collected in May 2017 in the Santa Luzia neighborhood, municipality of Codó, was positive for CHIKV, with a CT value of 27.9 and was identified as AR849404.

In the municipality of Caxias, two pools composed of a head and a body, respectively, of a single engorged *Ae. aegypti* female collected in March 2017 in the Campo de Belém neighborhood were positive for DENV-2, with CT values of 29.1 and 20.6, and identified as AR849486 and AR849487, respectively. 

### 3.3. Viral Isolation

Both AR849404 and AR849487 pools positive for CHIKV and DENV-2, respectively, where further submitted to viral isolation in cell culture and the infecting viral type was confirmed by the IIF test.

**Figure 2:**
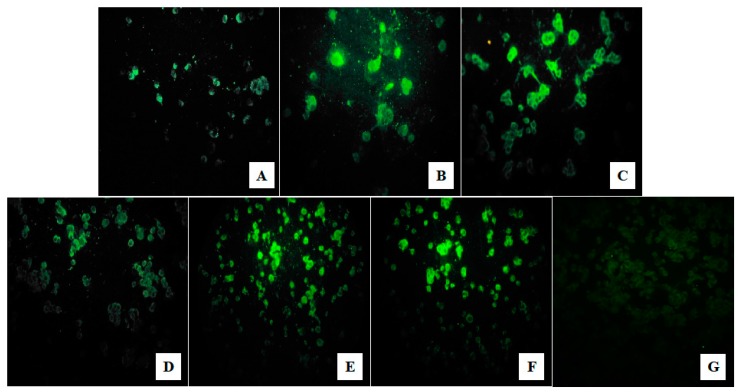
Indirect immunofluorescence test using *Alphavirus* and *Flavivirus* specific polyclonal antibodies to identify *Chikungunya virus* (CHIKV) and *Dengue virus* (DENV)-2 in C6/36 cell cultures. It is possible to observe a gradual increase in fluorescence (viral load) for both isolates. (**A**–**C**) for CHIKV and (**D**–**F**) for DENV-2 (in the first, second, and third successive passages, respectively). (**G**) shows uninfected C6/36 cells used as the negative control. Images at 40X magnification.

The pool AR849404, positive for CHIKV, did not show CPE during the cell passages, however, the IIF test was positive since the first inoculation, with a gradual increase in viral load over the passages, as shown in Figure 2A–C. The pool AR849487, positive for DENV-2, presented CPE only on the fifth day of the third passage, however, the IIF test was positive since the first inoculation, with a gradual increase in viral load during the passages, as shown in Figure 2D–F. 

### 3.4. Nucleotide Sequencing

**Figure 3:**
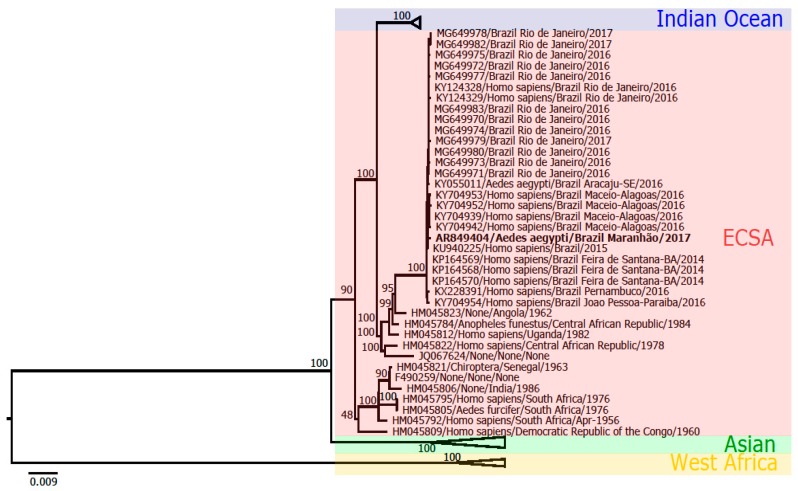
Maximum likelihood phylogenetic tree of CHIKV. The strain AR849404 (in bold) clustered within the CHIKV ECSA (East-Central-South African) genotype.

Phylogenetic analysis based on the complete genome sequencing of the sample AR849404 characterized the strain as belonging to the ECSA genotype, as shown in Figure 3, and it was deposited in GenBank, under access number MK518395.

**Figure 4:**
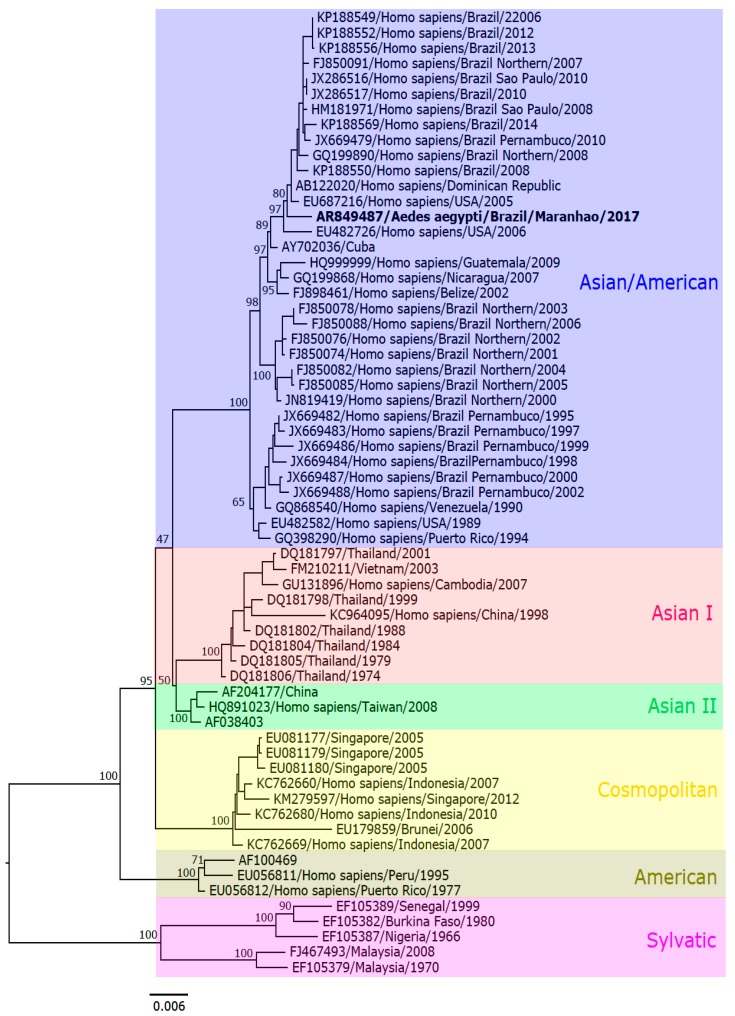
Maximum likelihood phylogenetic tree of DENV-2. The strain AR849487 (in bold) clustered within the DENV Asian/American genotype.

The complete genome sequencing of the sample AR849487 characterized the DENV-2 strain as belonging to the Asian/American genotype, as shown in Figure 4, which was deposited in GenBank, under access number MK517773.

## 4. Discussion

Despite the intensification of vector control programs as a measure to reduce the levels of *Ae. aegypti* and the transmission of arboviruses in Brazil, it has been shown that this vector is still widely spread nationwide, and in Maranhão, was found in the four cities studied, with a predominance in the municipality of Codó. This result reinforces other entomological studies carried out in the state, such as those in the municipalities of Caxias and São Luís, which have already registered the presence of *Ae. aegypti* throughout the year, with variations in its population indices [31,32,33,35]. Several aspects favor the proliferation of *Ae. aegypti*, being the predominant climatic factors, mainly due to the occurrence of rain [62,63]. However, in many municipalities of Northeast Brazil, especially in places with deficiency in the supply of water, the proliferation of *Ae. aegypti* occurs even in the months of low rainfall, due to the residents’ habit to store water for domestic activities in large deposits, such as tanks and barrels, providing continuous availability of breeding sites. This vector’s behavior has been reported in other studies performed in the municipalities of the interior of Maranhão [31,32,33,35,36].

A low number of *Ae. albopictus* mosquitoes were collected in this study and were only from the municipalities of Caxias and Codó, showing that this species still has a wild behavior, preferring areas with forests and vegetation. As this study was carried out in residences located in urban neighborhoods, few specimens were collected. However, it is necessary to monitor their density, as this species have been showing changes in behavior and were reported close to urban areas [64,65], including studies conducted in the municipality of Caxias, Maranhão [35,36]. 

The occurrence of *Ae. albopictus* arouses interest from health agencies, since, despite this vector having not been incriminated in the transmission of arboviruses in Brazil, adults and immature forms of this species have already been found naturally infected by DENV and ZIKV in Brazilian territory [66,67,68]. In addition, studies indicate the possibility of participation of Brazilian populations of this mosquito in the transmission of DENV, ZIKV, and CHIKV [69,70,71]. 

Another important fact is that *Ae. albopictus* is incriminated as the main vector of important arboviruses in different parts of the world, such as: DENV in Hawaii [72], China [73,74], Reunión [75], Gabon [76], and Mauritius [77]; ZIKV in Gabon [78]; and CHIKV in Reunión [75,79], Madagascar [80], and Gabon [76,81]. In that scenario, the possibility of this vector’s involvement in arbovirus transmission in Brazil cannot be ruled out.

Besides the collection of *Ae. albopictus* in urban areas during this study, other insects of medical importance were found, such as *Anopheles*, *Ceratopogonidae*, *Culicoides*, *Mansonia*, and *Uranotaenia*, which are atypical to domiciliary and peridomiciliary environments, showing a possible disorder in the habitats of those vectors in the region.

In recent decades, large areas of vegetation cover have been destroyed throughout the state of Maranhão. The municipality of Caxias, for example, lost large areas of vegetation for the construction of popular habitation sets and others with commercial purposes. This deforestation process favors the migration of mosquito vectors to urban areas, where they disperse and participate in the transmission of arboviruses [82,83].

In the present study, entomovirological surveillance detected the occurrence of specimens infected with CHIKV, belonging to the ECSA genotype, and DENV-2, belonging to the Asian-American genotype, which sustains epidemiological surveillance data on the circulation of those viruses in the region in humans [13,84,85,86]. Although this study has analyzed only *Ae. aegypti* and *Ae. albopictus*, the remaining insect species collected were stored and are available for further investigations.

CHIKV was detected in *Ae. aegypti* collected in Codó during 2017, however human infections have been reported in this municipality since 2016, when two cases were confirmed. In 2017, the number of chikungunya confirmed cases reached 46, showing that the virus has managed to establish and circulate in the region [84]. As many cases may be underreported during the arbovirus epidemics in Brazil, and as laboratory diagnosis may be troublesome in the interior of the state, entomovirological investigations play an important role in disease surveillance.

Chikungunya fever in Maranhão was first reported in September of 2014 and was related to an imported case [13], however, in January of 2016, the first autochthonous case of the disease was reported [85]. Moreover, only in that year, a total of 13,853 cases were reported. In 2017, a decrease in the number of notifications was observed (6416 cases), possibly reflecting the disease control actions [86]. 

CHIKV had already been detected in *Ae. aegypti* in the municipalities of Caxias and São Mateus do Maranhão, in an investigation performed during the first outbreak that occurred in the state in 2016, however, no information on the circulating genotype was available at that time [36]. 

In the present study, the ECSA genotype was identified, which corroborates other strains circulating in the northeast region of Brazil. The same genotype was detected in *Ae. aegypti* collected in Sergipe and represented the first report in Brazil and in the Americas [87], and in human samples from Alagoas, Bahia, Paraíba, and Sergipe [88,89,90]. 

Entomovirological investigations on *Ae. aegypti* in Brazil are restricted to few groups. The available data about CHIKV circulation are obtained from the human cases that show, until now, the circulation of the Asian genotype, in the Northern (state of Amapá [15]) and Southeast (Rio de Janeiro [91,92]) regions, and the ECSA genotype, reported in the Northern (Amazônia [93]), Northeast (Alagoas [88,89], Bahia [15,88,90], Paraíba [88], and Sergipe [90]), and Southeast (in Rio de Janeiro [90,94] and Minas Gerais [95]). 

The co-circulation of ECSA and Asian/Caribbean genotypes in Northeast Brazil has also been recently reported, including cases of simultaneous infection in clinical samples [96]. It can be inferred by our results, and those records in the literature in human samples, that the ECSA genotype is still prevalent in the country.

DENV-2 was also detected in *Ae. aegypti*, in mosquitoes collected in the municipality of Caxias in 2017, a result that corroborates the epidemiological information of the locality. In the same year, a total of 348 dengue cases were reported, with almost 90% of those (308) clinical-epidemiologically confirmed. In 2016, an increased number of cases was reported, and 1593 notifications and 520 confirmations were reported [84], characterizing a high incidence of dengue in the municipality.

Due to the limited DENV surveillance in vectors, information of the circulating DENV serotypes is scarce. In official reports by the Brazilian Ministry of Health (MoH), Maranhão was one of the few states that did not present information about circulating serotypes in 2017. Despite this, the detection of the DENV-2 in this study is consistent with the epidemiological information reported for the country in this period. According to the MoH, DENV-2 was predominant in Brazil in 2017 and corresponded to 54.3% of infections countrywide, differently from what has been reported from 2009 to 2016, when DENV-1 was the predominant serotype [97].

In Maranhão, DENV-2 was reported for the first time in 2001, in the capital of São Luís, and the first cases of hemorrhagic fever and deaths occurred in the following year [98]. Studies have associated the occurrence of more severe forms of the disease to infections by DENV-2 [99,100]. Moreover, the higher genetic variability of this serotype may contribute to the disease severity [101].

In this perspective, the DENV-2 circulation in the interior of the state draws attention to a possible increased disease severity in the region where it was detected. This scenario can occur throughout the country considering that a large part of the population is not immunized against this serotype, since DENV-1 was predominant in the country for eight years, until 2016 [97].

All four DENV serotypes have already been reported in the state of Maranhão and, according to the MoH, a total of 23,633 and 7049 probable dengue cases were reported between 2016 and 2017, with incidences of 339.8 and 101.4 cases per 100 thousand inhabitants, respectively. Furthermore, during those years, 15 dengue fatal cases were reported in the state [86]. 

In this study, the Asian/American DENV-2 genotype was characterized in the mosquitoes and corroborates the findings from human cases occurring in several other states, such as Piauí, bordering Maranhão [102], and in the states of Rio de Janeiro [103] and São Paulo, both located in the Southeast region of the country [104,105]. Investigations on the circulation of the arboviruses’ serotypes and genotypes per locality are greatly important, since it is known that some viruses may cause more severe clinical manifestations, especially in areas with simultaneous circulation of different viral species.

Arboviral infections are usually confirmed in patients by clinical criteria, combined to confirmatory laboratory tests. However, this approach results in the case confirmations mostly after the epidemic onset and therefore, on a more difficult disease control. 

It is important to emphasize that municipalities suffering from irregular water supply shall instruct the population on the correct water storage to reduce potential vectors’ breeding sites in endemic areas, such as the state of Maranhão.

Overall, this study contributes important information about the circulation of arboviruses of medical importance in the interior of the Maranhão, a transition area between the North and Northeast regions of Brazil, besides monitoring the dispersal of mosquito vectors occurring in urban areas. In that scenario, entomovirological surveillance is a useful tool for arboviral control programs, and may be effective to predict epidemics, confirm viral circulation, and provide information on the vectors involved in the transmission cycles, important for understanding the disease transmission dynamics.

## Figures and Tables

**Table 1:** Distribution of insects of medical importance collected in the municipalities of Alto Alegre do Maranhão, Caxias, Codó, and São Mateus do Maranhão, state of Maranhão during the investigation period.

Specification	Municipalities				Total	
	Alto Alegre do Maranhão	Caxias	Codó	São Mateus do Maranhão	N	%
*Aedes* spp.	6	0	1	0	**7**	**0.22**
*Aedes aegypti*	49	55	213	31	**348**	**10.99**
*Aedes albopictus*	0	1	11	0	**12**	**0.38**
*Aedes scapularis*	3	2	1	0	**6**	**0.19**
*Aedes taeniorhynchus*	0	0	2	0	**2**	**0.06**
*Anopheles (Nyssorhynchus)* sp.	0	0	1	0	**1**	**0.03**
*Ceratopogonidae*	1	0	0	0	**1**	**0.03**
*Culex (Culex)* spp.	3	0	0	0	**3**	**0.09**
*Culex* spp.	93	0	0	0	**93**	**2.94**
*Culex (Melanoconion)* spp.	2	0	2	0	**4**	**0.13**
*Culex quinquefasciatus*	713	238	1063	630	**2644**	**83.51**
*Culex spissipes*	0	13	0	0	**13**	**0.41**
*Culicoides paraensis* group	1	0	0	0	**1**	**0.03**
*Mansonia (Mansonia)* spp.	9	0	1	1	**11**	**0.35**
*Mansonia titillans*	2	0	0	0	**2**	**0.06**
*Phlebotominae*	11	2	2	1	**16**	**0.51**
*Uranotaenia hystera*	1	0	0	0	**1**	**0.03**
*Uranotaenia lowii*	0	0	1	0	**1**	**0.03**
**Total**	**894**	**311**	**1298**	**663**	**3166**	**100.00**
